# Stone Fruits: Growth and Nitrogen and Organic Acid Metabolism in the Fruits and Seeds—A Review

**DOI:** 10.3389/fpls.2020.572601

**Published:** 2020-09-25

**Authors:** Franco Famiani, Claudio Bonghi, Zhi-Hui Chen, María F. Drincovich, Daniela Farinelli, María V. Lara, Simona Proietti, Adolfo Rosati, Giannina Vizzotto, Robert P. Walker

**Affiliations:** ^1^Dipartimento di Scienze Agrarie, Alimentari e Ambientali, Università degli Studi di Perugia, Perugia, Italy; ^2^Department of Agronomy, Food, Natural Resources, Animals and Environment, University of Padova Agripolis, Legnaro, Italy; ^3^College of Life Science, University of Dundee, Dundee, United Kingdom; ^4^Facultad de Ciencias Bioquímicas y Farmacéuticas, Centro de Estudios Fotosintéticos y Bioquímicos, Consejo Nacional de Investigaciones Científicas y Técnicas, Universidad Nacional de Rosario, Rosario, Argentina; ^5^Istituto di Ricerca sugli Ecosistemi Terrestri, Consiglio Nazionale delle Ricerche, Porano (TR), Italy; ^6^CREA Centro di ricerca Olivicoltura, Frutticoltura e Agrumicoltura, Spoleto (PG), Italy; ^7^Department of Agricultural, Food, Environmental, and Animal Sciences, University of Udine, Udine, Italy

**Keywords:** endocarp metabolism, fruit growth, nitrogen compounds, organic acids, seed metabolism

## Abstract

Stone fruits of the Rosaceae family consist of several distinct parts, and these include the flesh, woody endocarp, and seed. To understand the metabolism of these fruits, it is necessary to have knowledge of both their structure and growth characteristics. The nitrogen metabolism of the different tissues of stone fruits is interlinked. For example, there is an import and storage of nitrogenous compounds in the endocarp that are then exported to the seed. Moreover, there are links between the metabolism of nitrogen and that of malic/citric acids. In this article, the structure and growth characteristics, together with the import/export, contents, metabolism, and functions of nitrogenous compounds and organic acids in the different parts of stone fruits and their seeds are reviewed.

## Introduction

The term stone fruits commonly refers to certain species of the genus *Prunus* which is a member of the rose family (*Rosaceae*) (Looney and Jackson, 2006). These fruits include almonds (*P. dulcis*), apricots (*P. armeniaca*), sweet cherries (*P. avium*), sour or tart cherries (*P. cerasus*), several species of commercial plums, the most important being the European plums (*P. domestica*) and the Asian or Japanese plums (*P. salicina*), peaches and nectarines (*P. persica*). The term stone fruits derives from the woody endocarp (stone or pit) which characterises the fruits of these species. The edible portion of stone fruits consists of the fleshy epicarp and mesocarp which encloses the stony endocarp. The exceptions are almonds and certain apricots whose seeds are consumed (Looney and Jackson, 2006). Botanically, the fruit of stone fruits is classified as a drupe.

Organic acids and nitrogenous compounds are abundant constituents of stone fruits and their seeds (Walker et al., 2011a; Famiani et al., 2015). Organic acids affect the taste of the flesh and skin of stone fruits and also have several underlying metabolic roles, which include both acting as an osmoticum that contributes to generating turgor pressure and links with nitrogen metabolism (Famiani et al., 2016; Walker and Famiani, 2018). Nitrogenous compounds play several roles in metabolism, and these include acting as the building blocks of proteins and serving as precursors that are used in the synthesis of compounds such as lignin; and the latter process is particularly pronounced in the endocarp (Walker et al., 2011a; Famiani et al., 2012). The endocarp is a specialized part of the pericarp: during development it becomes woody and gives rise to the stone which encloses and protects the seed (Romani and Jennings, 1971). The seeds of stone fruits are composed of a number of distinct tissues, including the seed coat which distributes imported assimilates to the developing internal storage tissues (Walker et al., 2011a; Famiani et al., 2012). The nitrogen metabolism of the flesh, endocarp, and seed are linked, and this is because there is an import of nitrogenous compounds into specific tissues of the fruit (e.g., endocarp), storage of this material, and subsequently export to other parts of the fruit and also to the seed (Walker et al., 2011a; Famiani et al., 2012).

In this article, the import, contents, metabolism, and functions of nitrogenous compounds and organic acids that are abundant in the flesh, endocarp, and seeds of stone fruits are reviewed. In addition, the development and structure of the whole fruit, endocarp, and seed are considered.

## In Order to Understand the Metabolism of Stone Fruits Their Structure and Growth Characteristics Must be Considered

### Structure and Growth Characteristics

**Figure 1:**
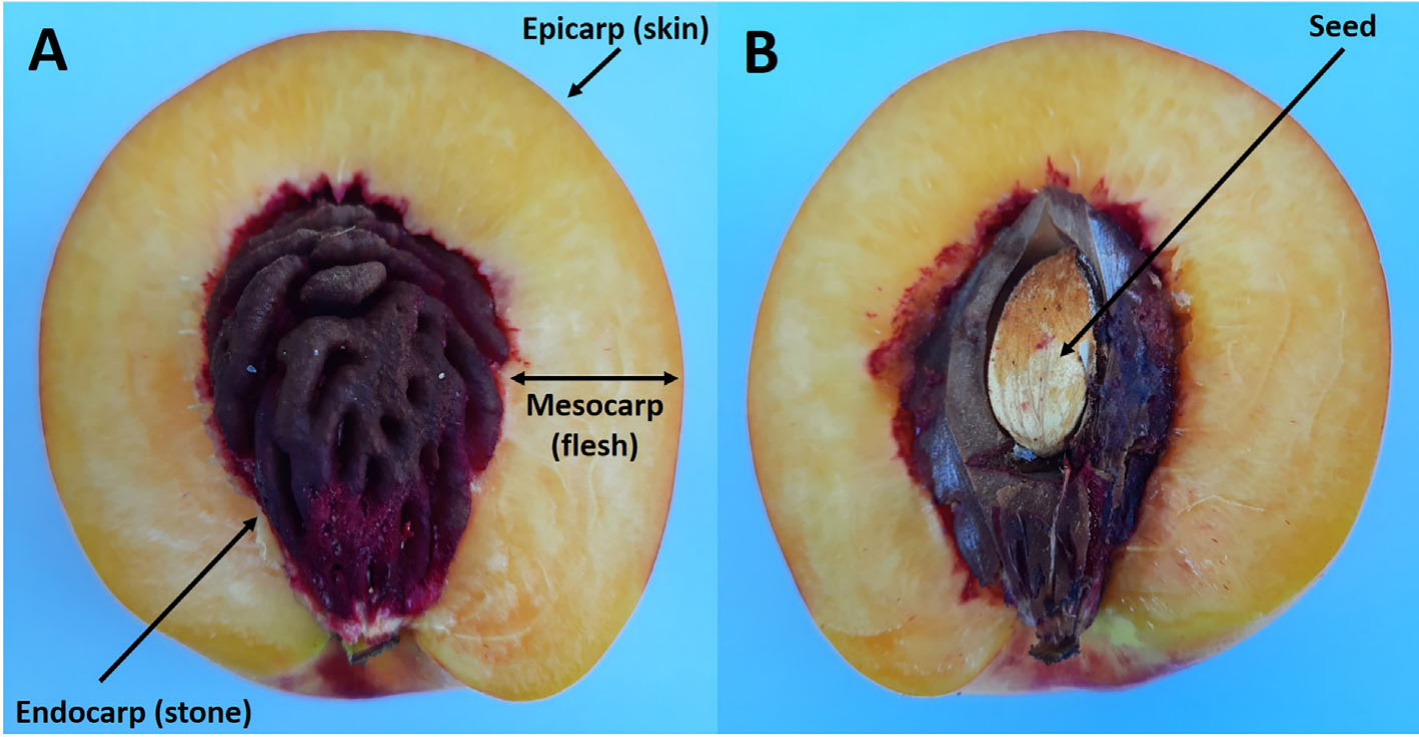
Longitudinal section of a ripe nectarine fruit showing the flesh and stone **(A)**. Same fruit but with the endocarp cut to reveal the enclosed seed **(B)**.

Stone fruits all consist of a fruit wall (pericarp) which usually encloses a single seed. The fruit wall is derived from the ovary and consists of three layers: the skin (epicarp) which encloses the flesh (mesocarp), and this then encloses the stone (endocarp) (**Figure 1**) (Romani and Jennings, 1971; Bollard, 1971; Brady, 1993). Recent studies suggest that simple genetic changes in a small number of genes that control development has brought about the evolution of the woody nature of the endocarp (Dardick and Callahan, 2014).

**Figure 2:**
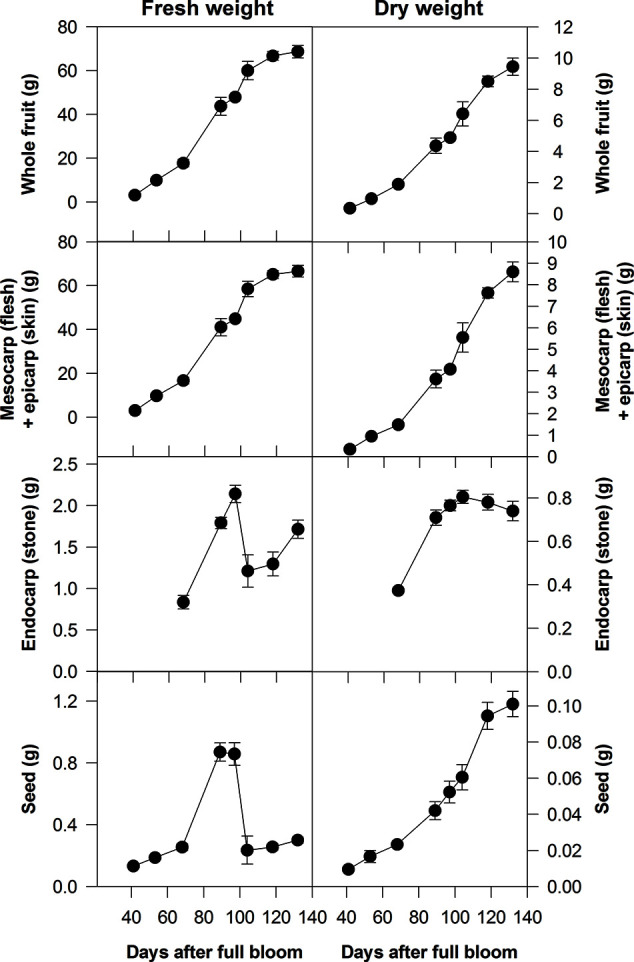
Fresh and dry weights of whole fruits of Ozark Premier plum and their component parts at different stages of development (from Famiani et al., 2012). Based on changes in their fresh weight, the growth patterns of whole fruits and their flesh plus skin were double sigmoidal: stage I was up to about 90 days after full bloom (DAB), stage III was after 100 DAB and stage II was between about 90 and 100 DAB. Bars represent the standard errors (n = 15).

On the basis of changes in either their fresh weight (FW) or volume, the growth pattern of either the whole fruit or the flesh can often be depicted by a double-sigmoidal curve. The first period of rapid growth as depicted by this curve is defined as stage I, the period of reduced growth is stage II and the second period of more rapid growth is stage III (**Figure 2**) (Lilleland, 1930; Lilleland, 1933; Pavel and DeJong, 1993b; Zuzunaga et al., 2001; Baldicchi et al., 2015). Stages I and III can be further subdivided. Thus, peach stage I can be subdivided into stages Ia and Ib and stage III can be subdivided into stages III and IV; with stage IV beginning when the growth rate of the flesh slows down and the fruit is close to its maximum size (Chalmers and van den Ende, 1975; Scorza et al., 1991; Zanchin et al., 1994). These subdivisions are advantageous when considering certain aspects of the growth and metabolism of peach fruits (Scorza et al., 1991; Tonutti et al., 1997). However, in this review, we use the traditional division into three stages, because these subdivisions have not been applied to all stone fruit species (**Figure 2**). In general, the following applies to all the stone fruits considered in this review. During stage I each part of the pericarp increases greatly in size and the stone and seed approach their maximum dimensions (size). During stage II the increase in the size of the flesh slows down, and the endocarp hardens to form the stone and it reaches its maximum dry weight (DW) (**Figure 2**). During stage III there is a large increase in the volume of the flesh and it ripens. In addition, the bulk of both the sugar content of the flesh and the storage reserves of the seeds are accumulated, and their DW increases (**Figure 2**) (Lilleland, 1933; Sterling, 1953; Marshall, 1954; Hawker and Buttrose, 1980; Bassi and Ryugo, 1990; Zanchin et al., 1994; Famiani et al., 2012; Falchi et al., 2013; Baldicchi et al., 2015). An exception is almond whose flesh does not expand during stage III (Hawker and Buttrose, 1980). The length of stage II depends on the variety of plum, peach, or cherry, and for some, it is very short, which gives rise to a sigmoidal pattern of growth (Lilleland, 1933; Pavel and DeJong, 1993a; Zuzunaga et al., 2001). Pavel and DeJong (1993b) evaluated the factors that could be responsible for the double sigmoidal pattern of growth and concluded that it might be the result of cultivar-specific developmental genetic information. By contrast, Dardick and Callahan (2014) proposed that the competition for assimilates between different parts of the fruit could be responsible for the double sigmoidal pattern of growth. More recently, these views have been perhaps reconciled by a study that compared Rosaceaeous fruits that showed either a single sigmoidal (pears) or double sigmoidal (peaches and strawberries) growth pattern (Pei et al., 2020). Thus, the block in fruit enlargement observed in peaches at stage II and in strawberry at the color-break stage is due to a diversion of assimilates and hormones toward endocarp lignification and anthocyanin biosynthesis, respectively. In contrast, the lack of dramatic changes occurring during pear fruit development allows the use of both hormones and assimilates for fruit enlargement, which resulted in the single sigmoid pattern. To support this view, a comparison of the three fruit transcriptomes during development allowed the identification of a set of genes differentially expressed in pears during the enlargement phase that are not expressed in the other two species. These genes included several transcription factors such as zinc finger proteins (ZFPs), which control cell size during plant organogenesis, and bHLHs (basic helix-loop-helix proteins), which regulate cell extension by transducing auxin signalling.

### Relative Growth of the Different Parts of the Fruit and Seed

In peach and apricot, fruit growth has also been analyzed in terms of the relative growth rate (RGR) of its different parts. In peach, this showed that the double-sigmoidal growth pattern corresponded to two phases of sink activity. During the first phase, RGR decreased logarithmically, while during the second RGR was relatively constant (DeJong and Goudriaan, 1989). The endocarp + seed had a higher RGR than the mesocarp during the first phase, while the RGR of the mesocarp was higher when the RGR of the whole fruit started to become relatively constant (Pavel and DeJong, 1993b). The transition between these two phases appeared to correspond to the beginning of sugar accumulation in the mesocarp and epicarp (Pavel and DeJong, 1993b). In this study of peach, the endocarp and the seed were considered as one entity; however, in apricot, the growth of the endocarp and different parts of the seed were each evaluated: thus making it possible to compare the growth rates of the different parts of the fruit (mesocarp + epicarp and endocarp) and seed (integuments and embryo + endosperm) (Baldicchi et al., 2015). In apricot, the endocarp had a higher RGR than the mesocarp + epicarp during stage I of growth; however, the RGR of the mesocarp + epicarp was also high (Baldicchi et al., 2015). During stage II (when stone hardening takes place) the embryo + endosperm had the highest RGR. This suggested that at this time, the strongest sink is the seed, which is the organ with the greatest ability to import assimilates (Baldicchi et al., 2015). During stage III of growth the RGR of both the epicarp + mesocarp and the embryo + endosperm had similar high values while the RGR of the endocarp was low (Baldicchi et al., 2015). During stages II and III, the high RGR of the seed (in terms of DW) was due to the import of assimilates used in the synthesis of storage compounds. Further, the daily growth rate of the different parts of the fruit and seed (which also depends on their size) were compared in order to calculate the amount of assimilates imported into each of them per day (Baldicchi et al., 2015). On per fruit basis, the amount of assimilates imported into the mesocarp + epicarp was high during stage I and very high during stage III; while in the endocarp, import was highest during stage II (Baldicchi et al., 2015). The lower values of seed daily growth rate, despite a higher RGR than the fruit during stage II, was due to its smaller size. However, in marked contrast to the epicarp + mesocarp, fat accounts for a large percentage of the DW of stone fruit seeds (Femenia et al., 1995). To synthesize this fat from imported sugars, a large amount of ATP and reductant is required, and this is produced from the metabolism of imported sugars. Hence, the daily growth rate of the seed would be higher if the amount of sugars used to produce this energy and reductant is taken into account. Similar considerations apply to the endocarp because this consists largely of lignin; again, a compound which requires large amounts of ATP and reductant for its synthesis.

### Changes in Cell Structure During Flesh Development and Dilution Effects

There are marked changes in the structure of the flesh of stone fruits during development (Scorza et al., 1991; Zanchin et al., 1994) and, in order to understand their metabolism, these must be taken into account. The increase in the size of the flesh results from both an increase in the number of parenchyma cells (brought about by cell division) and an increase in their size (arising from cell expansion), along with an increase in intercellular spaces (Masia et al., 1992). The contribution of each of these processes is dependent upon the stage of development. Stage I begins at full bloom and in peach it can be divided into stages Ia and Ib: in the flesh, the cell number increases 11.3 times and cell area 3.5 times during stage Ia, whereas cell number increases 1.1 times and cell area 9.2 times during stage Ib (Scorza et al., 1991; Masia et al., 1992). Similarly, in cherry, the bulk of cell division occurs during the first half of Stage I, and the greatest increase in cell size occurs during the second half of stage I (Tukey and Young, 1939).

In peach, fruit size is determined, at least in part, by the characteristics of the flower and particularly the ovarian tissues. Large-fruited peach cultivars produce ovaries and fruits with a higher number of cells than small-fruited cultivars (Scorza et al., 1991). The same is true for olive, which is also a drupe, where ovary size and cell number correlate with fruit size at maturity and the correlations hold even at the individual tissue (i.e., endocarp and mesocarp) level (Rosati et al., 2009; Rosati et al., 2012). By contrast, this is not true in cherry (*Prunus avium* L.). While fruit size in cherry is also associated with cell number and not with cell size, it is not correlated with the ovary size. Instead, differences in fruit size at maturity arise from a longer post-bloom cell division phase and are not related to ovary size and cell number at anthesis (Olmstead et al., 2007). Also in plum, differences in the number of cell divisions after anthesis are largely responsible for the genetic differences in fruit size among different species/varieties (Cerri et al., 2019). For both plant breeders and growers, it is important to know the mechanisms that influence fruit size; because in species in which fruit size is determined primarily by post-bloom processes, the possibility of altering fruit size (e.g., through irrigation or fertilization) is greater than in species where differences in fruit size are already determined at anthesis (Rosati et al., 2009; Cerri et al., 2019).

Although the number of parenchyma cells of the flesh is the major factor that determines the difference in potential fruit size among cultivars of both peach and cherry, environmental factors and cultural practices also affect the final size of the fruits of a given cultivar, largely as a result of an alteration in the size of the parenchyma cells of the flesh (Scorza et al., 1991; Zanchin et al., 1994; Olmstead et al., 2007). In peach flesh, the volume of individual cells increased nearly 200-fold during stage I, and from the end of stage I to ripeness about 20-fold (Zanchin et al., 1994); altough in absolute terms the increase in volume is greater during stage III. Clearly, this increase in cell size results in a large decrease in the cell wall area per g FW of flesh (g^−1^ FW). In peach, the expansion of the parenchyma cells during stage I was associated with an increase in intercellular spaces, and this was associated with the digestion of the middle lamella (Masia et al., 1992). Nevertheless, these cells remained connected by plasmodesmata, and the latter were grouped together to form pit fields (Zanchin et al., 1994). During stage I, there is a large increase in the proportion of total cell volume occupied by the vacuole (Famiani et al., 2012; Génard et al., 2014). The concentration of protein in these vacuoles is much less than in the cytoplasm and this results in a large decrease in the total soluble protein content of both the flesh and skin, as it is observed in both cherry and plum (Donen, 1939; Walker et al., 2011b; Famiani et al., 2012; Génard et al., 2014). In the parenchyma cells of tomato flesh, the change in the proportions of cell volume occupied by the vacuole and cytoplasm during development has been investigated in more detail: the large increase in the proportion of cell volume occupied by the vacuole occurs largely during the cell division stage of development, but also continues into the cell expansion stage (Beauvoit et al., 2014). This is consistent with the decline in total polypeptide abundance on SDS-PAGE gels of stone fruits flesh that is evident during development when gels are loaded with extracts of flesh (Walker et al., 2011b; Famiani et al., 2012). This change in the ratio of the cytoplasm to the vacuole can be a major factor that contributes to the changes in the abundance of individual enzymes per g FW of flesh during development. Thus, it is essential to take into account how enzyme abundance is expressed; because, if the content is expressed on a per g FW basis, different patterns of changes during development will be obtained than if expressed on per g total protein. Similarly, expansion of the flesh can lead to a decrease in the concentration of specific metabolites (g^−1^ FW) that is not brought about by a net catabolism of the metabolite but by a dilution effect (Famiani et al., 2015; Famiani et al., 2016; Moscatello et al., 2019). Similarly, when content is expressed on per g DW basis, a decrease during development could be a result of a dilution effect arising from the accumulation of large amounts of other material (for example sugars during ripening). Expressing content on a per fruit basis discriminates between these two possibilities (Famiani et al., 2015; Famiani et al., 2016; Moscatello et al., 2019). During the development of both peach and Japanese plum, the amount of CO_2_ released per unit time and per g of FW or DW (g^−1^ FW or g^−1^ DW) decreases greatly during stage I (Pavel and DeJong, 1993a; Zuzunaga et al., 2001), and a large part of this decrease could result from an increase in the proportion of cell volume occupied by the vacuole (Famiani et al., 2016). Indeed, the 3- to 5-fold decrease in CO_2_ release per unit time and g^−1^ FW or DW that occurs during stage I in stone fruit (Pavel and DeJong, 1993a; Zuzunaga et al., 2001) and the 4- to 5-fold increase in the proportion of cell volume occupied by the vacuole in tomato fruit during the equivalent stage of growth (Beauvoit et al., 2014) are consistent with this. The large decrease during development in the amount of cell wall area g^−1^ FW (Scorza et al., 1991) has also an effect on the abundance g^−1^ FW flesh of enzymes that are located in the cell wall. A further complicating factor is that the flesh and other tissues of fruit and seeds are composed of a number of distinct tissues, and many proteins and metabolites are not uniformly distributed between these tissues or between the different cell types that make up each of these tissues (Walker et al., 1999; Famiani et al., 2000; Walker et al., 2001; Famiani et al., 2016; Zanon et al., 2015).

## Import of Nitrogenous Compounds Into the Fruit and Seed and Related Aspects

### Import of Nitrogenous Compounds Into the Fruit and Seed

Almost all the nitrogenous compounds required by fruits are imported *via* the xylem and phloem of their vasculature (Layzell and LaRue, 1982; Peoples et al., 1985). During the growth of cowpea fruits 72% of nitrogenous materials are imported in the phloem, and the xylem provides the remainder (Peoples et al., 1985). In sweet cherry, the amide/amino acid content of the xylem was about 35–60 mM about 2 weeks after bud burst and declined to about 2-5 mM 2 months later (Grassi et al., 2002; Millard et al., 2006). In the xylem of terminal shoots of peach and Japanese plum (both growing in Florida, USA in July/August), this concentrations was 1–10 mM (Andersen et al., 1995a; Andersen et al., 1995b). Given the content of nitrogenous material in the ripe flesh of stone fruits (see below), and taking into account the amounts of liquid entering through the xylem and phloem (Morandi et al., 2007; Brüggenwirth et al., 2016), it would appear that the xylem can potentially supply a considerable proportion of the required nitrogenous compounds. However, this may not necessarily be the case because, in both soybean and wheat, there is a considerable transfer of nitrogenous compounds from the xylem to the phloem during their transport through the plant, and the input of nitrogenous compounds into the fruit is largely *via* the phloem (Layzell and LaRue, 1982: Simpson et al., 1983). In the xylem of both peach and sweet cherry, asparagine and glutamine account for a large proportion of the nitrogenous material (Andersen et al., 1995b; Grassi et al., 2002). Similarly, in the phloem of the shoot apex of peach, asparagine, glutamine, and glutamate accounted for the bulk of the amide/amino acid content (Moing et al., 1997). Both asparagine and glutamine are also abundant in the phloem and xylem of almond (Youssefi et al., 2000). Thus, in stone fruits, it appears likely that a large proportion of the nitrogenous compounds imported into the fruits and seeds consists of glutamine and asparagine.

### Changes in the Ratio of Contents of Non-Nitrogenous to Nitrogenous Compounds During Fruit and Seed Development

The mature flesh and mature seed of stone fruits contain a very different ratio of contents of non-nitrogenous to nitrogenous compounds, and for the flesh a ratio of 40–80:1 is typical, whereas for the seed the ratio is about 5:1 (Donen, 1939; Hawker and Buttrose, 1980; Famiani et al., 2012). Further, in the seeds of stone fruits, there is a considerable accumulation of lipids after the accumulation of nitrogenous compounds is almost complete (Hawker and Buttrose, 1980; Bassi and Ryugo, 1990; Walker et al., 2011a; Famiani et al., 2012). Thus, the ratio of sugars (also used in lipid synthesis) to nitrogenous compounds required by the seed changes during development. Fruits and seeds of other plants also require different ratios of these compounds, and potentially this can be achieved either by export of material or by adjusting the solute contents of the xylem and/or phloem saps, the latter being possible by the transfer of solutes between the xylem and phloem (Layzell and LaRue, 1982; Peoples et al., 1985). In stone fruits, export of nitrogenous material from the endocarp occurs. In both cherry and plum, vegetative storage proteins are accumulated at earlier stages of endocarp development and then decline to almost undetectable amounts (Walker et al., 2011a; Famiani et al., 2012).

### Source of the Nitrogenous Compounds That Are Imported Into the Fruit and Seed During Their Early Growth

Trees of stone fruits that flower before the leaves are developed are thought to utilize a considerable proportion of carbohydrates stored in the roots and crown for the early growth of their fruits (Keller and Loescher, 1989; Loescher et al., 1990; Flore and Layne, 1999). During dormancy, these reserves consist largely of starch and sucrose, but sorbitol becomes the most abundant soluble sugar when bud burst approaches (Keller and Loescher, 1989; Jordan and Habib, 1996; Flore and Layne, 1999). Similarly, during the early growth of both peach and cherry fruits, a large proportion of the imported nitrogenous compounds arises from mobilization of nitrogen compounds that are stored in the roots and crown, and not from uptake from the soil (Taylor and May, 1967; Rufat and DeJong, 2001; Grassi et al., 2002; Policarpo et al., 2002; Millard et al., 2006). In peach, the largest proportion of these reserves consists of soluble nitrogenous compounds, and arginine accounts for the bulk of these. The remainder of these reserves consists of proteins that include vegetative storage proteins (Taylor, 1967; Taylor and May, 1967; Gomez and Faurobert, 2002; Wisniewski et al., 2004). As in the case of carbohydrate reserves (Loescher et al., 1990; Flore and Layne, 1999), the amount of stored nitrogenous compounds in the crown and roots follows an annual cycle. Nitrogenous material is accumulated after the end of shoot and fruit growth, and export of nitrogenous compounds from senescing leaves makes an important contribution to this accumulation. In spring, the nitrogenous reserves are mobilized, and the amounts of stored nitrogenous material decrease both before and after bud burst (Taylor and May, 1967; Gomez and Faurobert, 2002). When both nitrogenous and carbohydrate reserves are mobilized, the soluble products of these reserves are transferred to the xylem, and then move along with water in the transpiration stream (Van Bel, 1990; Aubry et al., 2019). For example, in the case of carbohydrates in the xylem sap of cherry, their contents drop from 15 to 2–3 mg ml^−1^ after bud break (Loescher et al., 1990), and given the amount of water imported into cherry during this period (Brüggenwirth et al., 2016), this content is insufficient to meet the demands of the fruit. This implies that during this period of early growth of the fruits there must be a considerable transfer of sugars from the xylem to the phloem in order to increase the concentration of sugars entering the fruit, and this is also known to occur in some other fruits (Layzell and LaRue, 1982; Peoples et al., 1985: Simpson et al., 1983; Van Bel, 1990; Aubry et al., 2019). A similar mechanism could also be used for nitrogenous compounds. Unlike in some other plants (Aubry et al., 2019), in stone fruits, virtually nothing is known regarding either the sites of these transfers between xylem and phloem or the underlying molecular physiology.

## Import of Organic Acids Into the Fruit and Seed

It is generally thought that, in fleshy fruits, the bulk of the organic acid content of the flesh is synthesized from sugars (Walker and Famiani, 2018). The following shows that in the pericarp of stone fruits, this is almost certainly the case. Organic acids are the compounds that give rise to the bulk of the protons that determines the titratable acidity of fruits. Malic, citric, and quinic acids account for the main part of the organic acid content of the flesh of all stone fruits (Walker and Famiani, 2018). In both ripe cherry flesh and peach flesh, titratable acidity throughout development was close to the amount of protons that can be calculated to arise from the organic acids that are present (Girard and Kopp, 1998; Moing et al., 1998). This shows that the bulk of these must either be synthesized in the flesh or imported in the undissociated form. Organic acids dissociate in solution to give the organic acid anion and a proton(s). The degree of dissociation is dependent on the pKa of the proton donating group(s) of the acid in question. The pKa is the pH at which the proton donating group is 50% dissociated, and at one pH unit higher than this it is about 90% dissociated (malic acid pKa_1_ = 3.4, pKa_2_ = 5.3; citric acid pKa_1_ = 3.1, pKa_2_ = 4.8, pKa_3_ = 6.4; quinic acid pKa = 3.4). At the high pH of the phloem sap, these organic acids will be almost totally dissociated; hence, it is unlikely that a large proportion of the organic acid content is imported in the phloem. In the xylem of trees, the malate content is generally lower than 12 mM (Schell, 1997), and in both terminal shoots of peach and Japanese plum (growing in Florida in July/August), the organic acid content of the xylem was less than 3.5 mM, with malate accounting for most of it (Andersen et al., 1995a; Andersen et al., 1995b). Comparing the inflows of liquid from the xylem (about 0.28 g fluid g^−1^ FW day^-1^ for stage I in peach; Morandi et al., 2007), a potential malate content of 3.5 mM in the xylem and the content of malate in peach flesh (about 50 mM in peach around the end of stage I; Famiani et al., 2016), it is clear that a large proportion of the malate content could potentially be imported. However, the pH of the xylem is often in the range 4.5–5.5, and at this pH a substantial proportion of the organic acid content would be dissociated. Therefore, it would appear that, although a small proportion of malic acid could be imported from the xylem, the bulk of the content is synthesized within the fruit.

## Contents of Nitrogenous Compounds in the Flesh

In this review, only abundant nitrogenous compounds are considered. Donen (1939) reported the contents of nitrogenous compounds in the flesh, endocarp, and seed of Japanese plum during development. In the flesh, protein content decreased from about 10 mg g^−1^ FW during stage 1 to about 3 mg g^−1^ FW in ripe fruits. Similarly, protein abundance g^−1^ FW in the skin and flesh of cherry and plum and in peach flesh decreased greatly during stage I (Lombardo et al., 2011; Walker et al., 2011b; Famiani et al., 2012). Subsequently, from stage II to ripeness the change in protein content of peach flesh was much less (Lombardo et al., 2011; Famiani et al., 2016), and during ripening, total protein content decreased from 1.10 to 0.77 mg g^−1^ FW (Prinsi et al., 2011). The content of total protein in the flesh of common apricot decreased from 0.42–0.38 to 0.31 mg g^−1^ FW during ripening (D’Ambrosio et al., 2013; Zhang et al., 2017). In cherry flesh, but not in Japanese plum or peach, the total protein content decreased during ripening (Krishnan and Pueppke, 1990; Walker et al., 2011b; Famiani et al., 2012; Famiani et al., 2016). In cherry flesh, there was a large accumulation of a thaumatin-like protein during ripening which accounted for around 42% of the total soluble protein content (Fils-Lycaon et al., 1996). Similarly, large amounts of both a thaumatin-like protein and lipid-transfer proteins are present in ripe peach flesh, and these can produce an allergic reaction in susceptible individuals (Palacin et al., 2010). These abundant proteins serve as a store of nitrogen and could potentially have other roles such as in plant defence and responses to stresses (Wisniewski et al., 2004; Tian et al., 2007; Dagar et al., 2010). The content of amides and amino acids can be increased greatly in the flesh of stone fruits by feeding the trees with nitrogenous fertilizer (Taylor, 1967; Jia et al., 2000). It is likely that the content of protein (and especially those that act as a store of nitrogen) will also be increased, as occurs in other fruits (Delgado-Alvarado et al., 2007: Famiani et al., 2012).

Asparagine accounts for a large proportion of amide/amino acid content of the flesh of plums, apricots, cherries, and peaches, and also in the endocarp and seeds of stone fruits species in which it has been studied (Kakiuchi et al., 1985; Famiani et al., 2012; Rodriguez et al., 2019). The amount of non-protein nitrogen present in Japanese plum flesh (Donen, 1939), would be equivalent to about 7.5 mg g^−1^ FW (stage I) and 3.5 mg g^−1^ FW (ripe flesh) of asparagine being present. In Japanese plum, flesh ammonium content was highest toward the end of stage I (0.2–0.3 mg g^−1^ FW), which accounted for 10–15% of the total N content of the flesh, and then decreased (Donen, 1939). Similarly, in the flesh of one cultivar of Japanese apricot, large amounts of ammonium accumulated up to ripening and then declined; this accumulation of ammonium was not observed in another cultivar (Otoguro and Kaneko, 1994). During the ripening of peach flesh, total amide/amino acid content increased from 1 to 1.3 mg g^−1^ FW (Prinsi et al., 2011).

In ripe peach flesh, the total amide/amino acid content of fruits from trees fed low amounts of nitrogenous fertilizer was 1.3 mg g^−1^ FW (0.8 mg of this was asparagine), and from trees fed larger amounts of nitrogenous fertilizer it was 4.6 mg g^−1^FW (2.6 mg was asparagine) (Jia et al., 2000). Moing et al. (1998) found that asparagine accounted for the bulk of the amino acid/amide content of peach flesh throughout development. In ripe peach flesh from three cultivars, the content of asparagine was between 3.2 and 4.7 mg g^−1^ FW and this accounted for over 85% of the amide/amino acid content (Moing et al., 2003). In the ripe flesh of *Prunus davidiana* (a related species to peach), asparagine content was about 9.5 mg g^−1^FW and accounted for over 90% of the amide/amino acid content (Moing et al., 2003). In Japanese apricot, asparagine also accounted for about 90% of the soluble nitrogenous compounds of the flesh throughout development. Asparagine (mg g^−1^ FW) content changed during development and was 4.5 in stage I, 7.0 in stage II, and 3.2 in stage III (ripeness) in 1982 season, and 3.0 in stage I, 5.6 in stage II, and 2.5 in stage III (ripeness) in 1983 season (Kakiuchi et al., 1985). Comparable amounts and patterns of changes in asparagine content were also found in two cultivars of small Japanese apricot (Otoguro and Kaneko, 1994). In cherry flesh, amide/amino acid content decreased from roughly 9.7 to 4.4 mg g^−1^ FW during ripening (Prinsi et al., 2016).

## Contents of Organic Acids in the Flesh

In this review, only the quantitatively most important organic acids are considered. For previous reviews dealing with organic acids in stone fruits and additional details, refer to Famiani et al. (2015) and Walker and Famiani (2018). In stone fruits, the most abundant organic acids are usually malic, citric, and quinic (Walker and Famiani, 2018). The contents of these acids per g FW differ between the species and cultivar of stone fruits and is also dependent on the tissue and stage of development (**Table 1**; Walker and Famiani, 2018). In the flesh of apricots and peaches both malic and citric acids can be abundant (Moing et al. 1998; Baldicchi et al. 2015; Famiani et al. 2016), whereas in the flesh of cherries and plums, malic acid is much more abundant (Walker et al., 2011b; Famiani et al., 2012; Moscatello et al., 2019). In most stone fruits the contents of malic/citric acid per g FW (i.e., concentration: mg g^-1^ FW) of both skin and flesh, are usually higher before stage III and then decrease during stage III. However, this decrease is at least in part due to a dilution effect which arises from cell expansion (fruit growth), and in some cases there is no net dissimilation of the acids (Famiani et al., 2015; Walker and Famiani, 2018). In order to establish if net dissimilation of the acids occurs, it is necessary to express their contents at different stages of development on a per fruit basis (i.e., mg fruit^-1^). Doing this, it was observed that in cherry flesh, the decrease in the content of malic acid per g FW during ripening, and up to the time of commercial harvesting, was due to a dilution effect; because its content per fruit increased for the whole period. Thus, there was net synthesis and not dissimilation of malic acid (Walker et al., 2011b). In apricot flesh, during the first part of ripening the decrease in both malic and citric acids per g FW was due to the growth of the fruit, because their contents per fruit increased, and a net dissimilation of these compounds only occurred in the last part of ripening (Baldicchi et al. 2015). A similar behavior was observed in the fruits of the plum cultivar Ozark premier (*P. salicina*) (Famiani et al., 2012). Up to commercial harvest the decrease in the concentration of malic acid in the flesh of the plum species/cultivars President (*P. domestica*), Shiro (*P. salicina*) and Mirabolano (*P. cerasifera*) was due to a dilution effect, and the amounts per fruit increased; after this time a net dissimilation of malic acid occurred in the over-ripe fruits (Moscatello et al., 2019). In peach flesh, during ripening, there was a net dissimilation of citric acid, whereas there was a synthesis of malic acid (Famiani et al. 2016). Therefore, during ripening there can be either a net dissimilation or synthesis of stored Krebs cycle acids, and which occurs is dependent on the species/cultivar and on the stage of ripening. The metabolism and functions of quinic acid in fruits (including stone fruits) has been recently reviewed by Walker and Famiani (2018), and hence, this topic is omitted in the present review.

**Table 1:** The concentration of organic acids (mg g^-1^ FW) in the flesh of unripe and ripe stone fruits.

	Unripe (stage II)			
	Malic acid	Citric acid	Total soluble sugars	
Apricot (common)	33	1.5	6.6	Baldicchi et al., 2015
Apricot (Japanese)	34	13	6.6	Otoguro and Kaneko, 1994
Cherry (sweet)	8	low	41	Walker et al., 2011b
Peach Hakuto			33	Moriguchi et al., 1990
Peach Adriatica	6.6	4.8	43	Famiani et al., 2016
Plum (Japanese)	17	0.1	35	Donen, 1939
				Famiani et al., 2012
	**Ripe (end of stage III)**		
	**Malic acid**	**Citric acid**	**Total soluble sugars**	
Apricot (common)	8	10	92	Baldicchi et al., 2015
Apricot (Japanese)	21	40	12	Otoguro and Kaneko, 1994
Cherry (sweet)	6.7	low	126	Ballistreri et al., 2013
				Walker et al., 2011b
	9.4	0,2	165	Winkler and Knoche, 2018
Cherry (sour)	21.6	0.3	115	Winkler and Knoche, 2018
Cherry (Morello)				
Line 16	35	low	171	Proietti et al., 2019
Line 37	37	low	55	
Peach Hakuto			72	Moriguchi et al., 1990
Peach Adriatica	4.8	0.9	68	Famiani et al., 2016
Peach Pamirskij	3	3	141	Moing et al., 2003
Nectarine Summergrand	4.3	3.7	113	Moing et al., 2003
Plum (Japanese)	11	0.1	104	Donen, 1939
				Famiani et al., 2012
*Prunus davidiana*	15	0.3	17.5	Moing et al., 2003

## Enzymes of Nitrogen and Krebs Cycle Acid Metabolism in the Flesh

**Figure 3:**
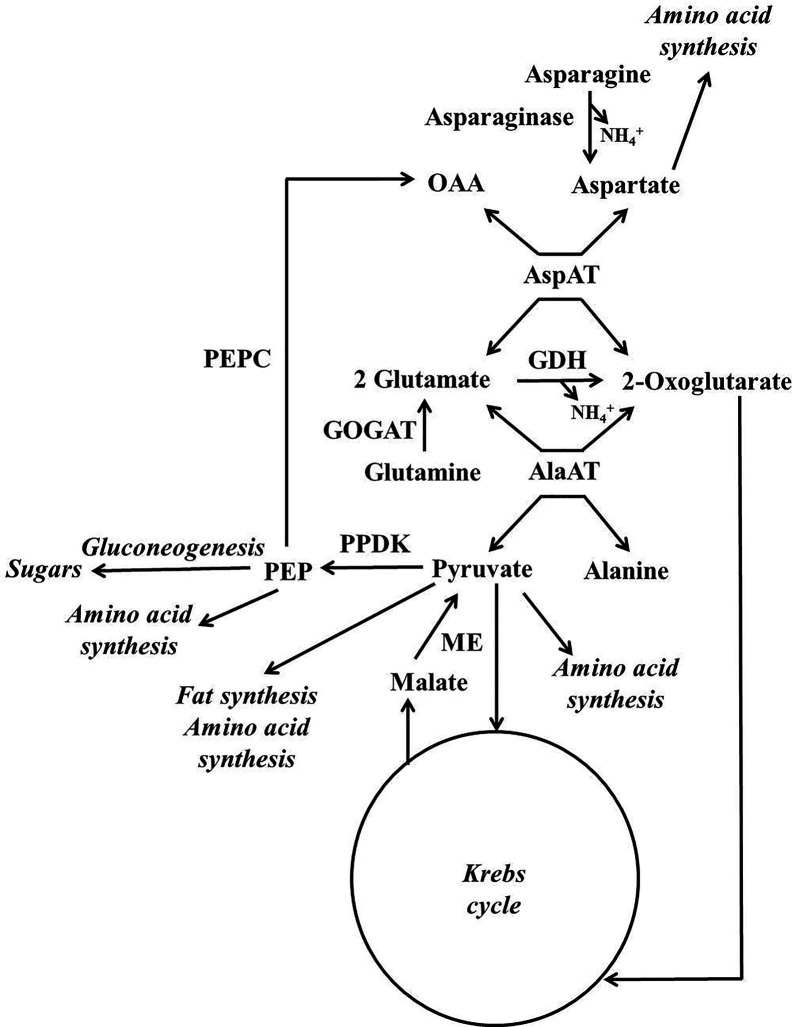
Simplified scheme outlining the major pathways involved in the metabolism of nitrogenous compounds and malic and citric acids in fruits and seeds of stone fruits (based on Walker et al., 2018). AlaAT = alanine aminotransferare; AspAT = aspartate aminotransferare; GDH = glutamate dehydrogenase; GOGAT = glutamate synthase also known as glutamine oxoglutarate aminotransferase; ME = malic enzyme; OAA = oxalacetate; PEP = phosphoenolpyruvate; PEPC = phosphoenolpyruvate carboxylase; PPDK = pyruvate orthophosphate dikinase.

**Figure 4:**
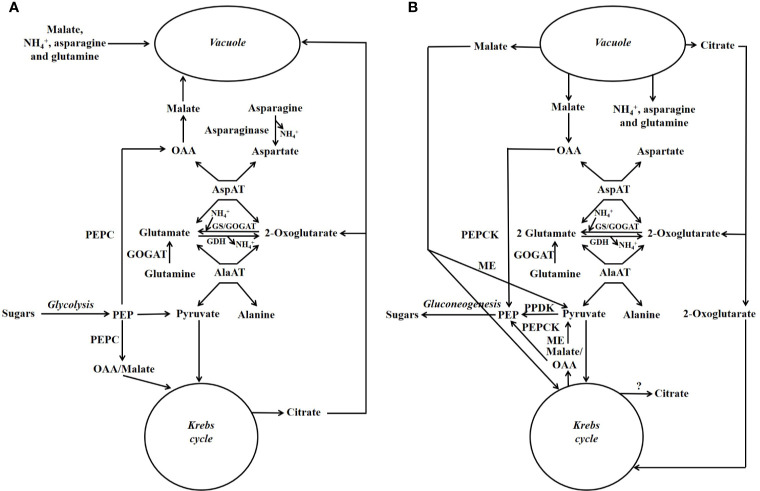
Simplified scheme outlining the major pathways involved in the metabolism of nitrogenous compounds and malic and citric acids in fruits and seeds of stone fruits (based on Walker et al., 2018). Unlike in **Figure 3**, the temporal organization of metabolism is taken into account. **(A)** Storage phase. **(B)** Utilization phase. GDH = glutamate dehydrogenase; GOGAT = glutamate synthase also known as glutamine oxoglutarate aminotransferase; GS = glutamine synthetase; ME = malic enzyme; OAA = oxalacetate; PEP = phosphoenolpyruvate; PEPC = phosphoenolpyruvate carboxylase; PEPCK = phosphoenolpyruvate carboxykinase; PPDK = pyruvate orthophosphate dikinase.

A scheme illustrating the position in metabolism of the enzymes mentioned in this section is given in **Figures 3**, **4**. Evidence has been provided that in both stone fruits and various other tissues of plants, malate and citrate, which are stored in the vacuole, can be released at certain situations, and then serve as a substrate for nitrogen metabolism (Walker et al., 2015; Famiani et al., 2016; Bräutigam et al., 2017; Walker et al., 2018; Walker and Famiani, 2018). To more fully understand this area of metabolism, it is essential to take this into account (Walker et al., 2018). The schemes depicted in **Figure 4A** show events occurring when malate and/or citrate are accumulated in the vacuole, and **Figure 4B** depicts events when they are released. When the amount of malate and/or citrate released from the vacuole is in excess of demands of processes other than gluconeogenesis, the latter occurs.

For simplicity in this review, we refer to malate and citrate as Krebs cycle acids, and this is because they are associated with the Krebs cycle. The metabolism of malate and citrate is intimately linked with that of amino acids and amides (**Figures 3**, **4**), and one reason for this is that the carbon skeletons of many nitrogenous compounds are synthesized from these organic acids (Walker and Famiani, 2018). Asparagine and glutamine appear to account for the bulk of the nitrogenous compounds imported into the fruits and seeds of stone fruits, however, little glutamine is present in the flesh, whereas large amounts of asparagine are present. Thus, it appears that there is a preferential utilization of glutamine in stone fruits, as also seen in some other plant tissues (Walker et al., 2011a; Famiani et al., 2012). An inspection of the data given in the section “contents of nitrogenous compounds in the flesh”, reveals that asparagine often accounts for over 50% of the protein and non-protein nitrogenous compounds present in the ripe flesh of stone fruits. Nevertheless, imported glutamine and asparagine must be converted to the spectrum of amides and amino acids required by the fruit and seed (Walker et al., 2011a; Famiani et al., 2012). The potassium-dependent asparaginase is the principle route by which asparagine is further metabolized in sink tissues (**Figures 3**, **4**; Lea et al., 2007). The enzyme glutamate synthase also known as glutamine oxoglutarate aminotransferase (GOGAT) converts glutamine to glutamate and is the main pathway used in the breakdown of glutamine (**Figures 3**, **4**; Lea, 1993). *In vivo*, glutamate dehydrogenase (GDH) catalyzes the deamidation of glutamate to 2-oxoglutarate (Lea, 1993). The enzymes glutamine synthetase (GS) and GOGAT acting in concert are responsible for the incorporation of ammonium into organic forms of nitrogen. The enzymes aspartate aminotransferase (AspAT) and alanine aminotransferase (AlaAT) catalyze reversible reactions which occupy a central role in nitrogen metabolism (**Figures 3**, **4**).

Phosphoenolpyruvate carboxylase (PEPC) is a key enzyme of both Krebs cycle acid and nitrogen metabolism, and it is a component of the predominant pathway used in the synthesis of malate in most plant tissues. PEPC catalyzes the carboxylation of phosphoenolpyruvate to oxaloacetate, whereas phosphoenolpyruvate carboxykinase (PEPCK) catalyzes the reverse reaction (Leegood and Walker, 2003). There is evidence that in sweet cherry phosphorylation of both these enzymes contributes to the coordinate regulation of PEPC and PEPCK and hence flux between PEP and OAA (Walker et al., 2016). Similarly, in peach flesh, it is possible that phosphorylation contributes to the regulation of PEPC activity (Moing et al., 2000). In addition, in peach flesh (unlike both cherry and plum flesh) PEPC is present as a polypeptide doublet, and this raises the possibility that PEPC ubiquitination also contributes to its regulation (Famiani et al., 2016).

In plants, two pathways can be used in the conversion of Krebs cycle acids and the carbon skeletons of many amino acids/amides to sugars, and one utilizes PEPCK, whereas the other utilizes pyruvate orthophosphate dikinase (PPDK) (Leegood and Walker, 2003). In the flesh of peach, both PEPCK and PPDK are present (Walker and Chen, 2002; Borsani et al., 2009; Lara et al., 2009; Lara et al., 2010; Famiani et al., 2012; Famiani et al., 2016). However, it would appear that PEPCK is many times more abundant than PPDK in both peach flesh and that of other stone fruits, and this implies that the bulk of any gluconeogenic flux utilizes the PEPCK pathway (Famiani et al., 2016). NADP-malic enzyme, which converts malate to pyruvate, is also present in the flesh of peach, plum, and cherry (Borsani et al., 2009; Walker et al., 2011b; Famiani et al., 2012; Famiani et al., 2016). PPDK, which converts pyruvate to PEP, is required to convert pyruvate produced by NADP-ME to sugars by gluconeogenesis. However, the low abundance of PPDK in the flesh of stone fruits implies that the bulk of pyruvate produced by NADP-ME is used by processes other than gluconeogenesis such as the Krebs cycle (Famiani et al., 2016).

In peach, a recent study found that both metabolism and storage in the vacuole contributed to the amount of malate that was accumulated, while metabolism was crucial for citrate accumulation. In particular, low-acid cultivars showed higher citrate degradation and less transport of malate into the vacuole, and this was due to up- and down-regulation of a GABA (γ-aminobutyric acid) pathway gene and a malate transporter gene, respectively (Zheng et al., 2021).

For a detailed description of pathways regarding malate and citrate synthesis and breakdown refer to Famiani et al. (2015) and Walker and Famiani (2018). In the flesh of sweet cherry, Japanese plum and peach, cytosolic GS, cytosolic AspAT, GDH, asparaginase, PEPC and PEPCK are present throughout development. Similarly, at the time when there is an extensive metabolism of nitrogenous compounds, these enzymes are also abundant in the developing seed and endocarp of sweet cherry and Japanese plum (Moing et al., 2000; Lombardo et al., 2011; Walker et al., 2011a; Famiani et al., 2012; Rodriguez et al., 2019).

## Functions of Krebs Cycle Acids

In addition to a direct role in metabolism, the Krebs cycle acids also have other functions. In the flesh of stone fruits, the ratio of organic acids to sugars is much higher in unripe as compared to ripe fruits (**Table 1**). This high ratio is thought to contribute to making the flesh less palatable, and this protects the developing fruits and seeds from animals (Walker and Famiani, 2018). The Krebs cycle acids are also important in turgor regulation. In tomato flesh, malate is an important osmoticum and is thought to play a key role in generating turgor pressure to drive cell expansion (Guillet et al., 2002; Beauvoit et al., 2014). At certain stages of the development of the flesh of some stone fruit species such as apricot, malic and citric acids can be more abundant than sugars (Baldicchi et al., 2015), and clearly, these acids are a major osmoticum. The sugar content of Japanese apricot flesh is quite low throughout development and citric acid is more abundant (Otoguro and Kaneko, 1994); hence, citric acid can be a predominant osmoticum even in ripe fruit. In carrot tap root, the relative contributions of organic acids and sugars for generating cell turgor pressure vary according to the position in the root (Korolev et al., 2000) and, similarly, both sugars and malate can contribute to turgor pressure changes in stomata that bring about stomatal movement (Talbott and Zeiger, 1996). Thus, it is clear that either organic acids or sugars can be the major osmoticum used to generate cell turgor, and which is used depends on a number of factors that include species, cell type, and stage of development.

## Endocarp Metabolism

During stage I of development, the endocarp increases greatly in size, and then, during stage II, the endocarp hardens to form the stone. There are marked changes in the structure of the parenchyma cells of the endocarp during development (Masia et al., 1992). At both 1 and 4 weeks after full bloom, these cells contained vacuolar inclusions of a phenolic nature. These inclusions were absent in the mesocarp, and it was hypothesized that they might contain precursors used in lignin synthesis. These inclusions disappeared by the middle of stage II (8 weeks after full bloom), and at this time, the cell walls were lignified and contained numerous simple pits containing cytoplasmic channels (Masia et al., 1992).

The endocarp of Japanese plum during stage I contained about 7 mg g^−1^ FW each of sucrose, glucose, and fructose. During stage II, the contents of glucose and fructose decreased and during stage III, the values of these sugars, together with that of sucrose, increased to 3–10 mg g^−1^ FW (Famiani et al., 2012). The sucrose content of cherry endocarp is low throughout development, and the contents of glucose and fructose are in the range 2–14 mg g^−1^ FW and show a similar pattern of changes during development to that observed in Japanese plum endocarp (Walker et al., 2011a; Famiani et al., 2012). In both Japanese plum and sweet cherry, the decrease in soluble sugar contents during stage II occurred at the same time as the massive rate of increase in endocarp DW, and the increase in soluble sugar contents during stage III occurred when this rate of DW increase slowed, altough some of this latter increase could also result from contamination of endocarp by the flesh/juice which, at this time, contain large amounts of soluble sugars (Walker et al., 2011a; Famiani et al., 2012). In peach endocarp during stage I, sucrose, sorbitol, and starch contents were <1.3 mg g^−1^ FW and glucose/fructose contents were 7–18 mg g^−1^ FW (Lo Bianco and Rieger, 2002). In peach endocarp during stage I, the activity (µmol g^−1^ FW h^−1^) of sucrose synthase (SuSy) was 1.0, acid invertase was 6.5 and alkaline/neutral invertase was 0.5 (Lo Bianco and Rieger, 2002). Either invertase or Susy is required for the breakdown of sucrose in most plant tissues (Kingston-Smith et al., 1999). Hu et al. (2011) reported that a number of enzymes, including several involved in the glycolytic pathway, decreased in peach endocarp after 28 DAB; however, this decrease could be misleading because results were expressed on a per DW basis, and there is a massive increase in endocarp DW arising from lignification.

There is a massive and transient accumulation of vegetative storage proteins in the endocarp of both sweet cherry and Japanese plum. Enzymes involved in the metabolism of nitrogenous compounds (e.g., GS, cytosolic AspAT, and PEPC) are abundant at the time when these storage proteins are accumulated and subsequently mobilized. The accumulation of these storage proteins is likely associated with the storage of excess imported nitrogenous compounds; because the endocarp requires a very large amount of sugars and not nitrogenous compounds to fuel lignin synthesis (Walker et al., 2011a; Famiani et al., 2012). In stone fruits, export of nitrogenous material from the endocarp occurs; thus, in both cherry and plum, vegetative storage proteins are accumulated at early stages of endocarp development, and subsequently decline to almost undetectable amounts (Walker et al., 2011a; Famiani et al., 2012). This decline coincided with storage protein accumulation in the seed (Walker et al., 2011a; Famiani et al., 2012). In addition, the total N content of the endocarp of plum declines greatly as the fruit matures, and it can be calculated that this decline in the content of nitrogenous material is equivalent to about 35% of the nitrogenous compounds accumulated in the seed (Donen, 1939; Famiani et al., 2012). In Japanese apricot, asparagine accounted for about 60–80% of the soluble nitrogenous compounds of the endocarp throughout development. Asparagine (mg g^−1^ FW) content changed during development: 5.0 in stage I, 3.3 in stage II, and 1.3 in stage III (ripeness) in 1982 season, and 3.2 in stage I, 1.7 in stage II, and 0.8 in stage III (ripeness) in 1983 season (Kakiuchi et al., 1985). In the endocarp, nitrogen metabolism is also associated with lignin synthesis. This is because massive amounts of ammonium are released during this process by the action of phenylalanine ammonium lyase (Lea, 1993). The presence of enzymes involved in nitrogen metabolism in endocarp is also likely related to the reassimilation of this ammonium. The occurrence of PEPCK in the developing endocarp (Walker et al., 2011a; Famiani et al., 2012; Walker et al., 2018), raises the possibility that (as described in **Figure 4**) ammonium could accumulate in the vacuole and then, sometime later, could be released and re-assimilated.

## Seed Metabolism

The principal tissues of the seeds of stone fruits are the seed coat (integuments), nucellus, endosperm, and embryo. The whole seed, integuments, and nucellus reach their final size during stage I of development. During the early part of stage II, the endosperm grows and largely replaces the nucellus, while later in stage II, the embryo grows and replaces much of the endosperm (Marshall, 1954). The growth of the different parts of the seed follows a sigmoidal pattern; however, the growth of the whole seed is double-sigmoidal, and this is because the growth of the different parts is asynchronous (Chalmers and van den Ende, 1975; Bassi and Ryugo, 1990). During development, the nucellus and endosperm serve as temporary stores for small amounts of imported assimilates (Hawker and Buttrose, 1980; Bassi and Ryugo, 1990). Most of the storage reserves in the mature seeds are located in the embryo, and the bulk of these are only deposited after the embryo approaches its maximum size (Lilleland and Newsome, 1934; Tukey and Lee, 1937; Hawker and Buttrose, 1980; Walker et al., 2011a; Baldicchi et al., 2015). The storage reserves in the mature embryo consist largely of lipids (c300-420 mg g^−1^ FW), storage proteins (c120 mg g^−1^ FW) and smaller amounts of soluble sugars (20–70 mg g^−1^ FW). Very low amounts of starch are present in the mature seed (Tukey and Lee, 1937; Hawker and Buttrose, 1980; Bassi and Ryugo, 1990; Lo Bianco and Rieger, 2002; Walker et al., 2011a; Famiani et al., 2012).

The seed coat of stone fruits possesses a well-developed vasculature, and it is likely that imported material is distributed throughout the seed coat in the phloem and then unloaded and transported to the developing storage tissues (Hawker and Buttrose, 1980). Similarly, in some other seeds, the seed coat plays a key role in the distribution of imported materials (Walker et al., 1999; Patrick and Offler, 2001). In these seeds, it appears that water and assimilates exit the phloem and enter the parenchyma cells of the seed coat by bulk flow through plasmodesmata, which is driven by the high hydrostatic pressure of the phloem (Patrick and Offler, 2001). However, recent studies have indicated that the hydrostatic pressure in the phloem in unloading zones could be much lower than previously thought (Ross-Elliott et al., 2017; Milne et al., 2018). In the seed coat of grape, cell wall invertase is abundant in the palisade layer, a tissue which is thought to transport imported nutrients to the developing storage tissue, and whose cells are connected by numerous plasmodesmata (Walker et al., 1999; Famiani et al., 2000). In this tissue, cell wall invertase could contribute to the regulation of the turgor pressure of the parenchyma cells by increasing the solute concentration in the apoplast, and thus may function in contributing to maintaining a decreasing turgor pressure gradient between the phloem and sink cells that allows a symplastic flow. The seeds of stone fruits approach their maximum FW and size before the bulk of their storage materials are accumulated (Lilleland and Newsome, 1934; Tukey and Lee, 1937; Hawker and Buttrose, 1980; Walker et al., 2011a; Famiani et al., 2012; Baldicchi et al., 2015). Thus, water and large amounts of assimilates must be imported into the seed after it approaches its maximum size. It is possible to estimate the amount of water required for the import of the assimilates necessary for the synthesis of these storage materials. This can be done by comparing the amount of dry matter accumulated in the seed with the dry matter content of the phloem. Thus, in the case of one almond seed about 1,200-mg dry matter are accumulated (Hawker and Buttrose, 1980). For peach and sweet cherry the dry matter content of the phloem sap is about 160–180 mg dry matter ml^−1^ (Morandi et al., 2007; Brüggenwirth et al., 2016). This means that about 8 ml of water is needed to import this material, and this excess water must be removed from the seed. In the case of one apricot seed about 600-mg dry matter are accumulated (Baldicchi et al., 2015) and, using the same figure for the concentration of assimilates in the phloem, this would require about 4 ml of water. In apricot, the FW of seed coat about midway through the period of storage deposition in the embryo is about 0.1 g per seed, and the bulk of the storage reserves are deposited over a period of 50 days. Thus, if 4 ml of water pass through the seed coat during these 50 days, then on average at least 0.08 ml of water passes through the coat each day, and this is similar to the FW of the coat (Baldicchi et al., 2015). The most likely fate of this excess water is that, as in seeds in which it has been studied, it is exported in the xylem (Pate et al., 1985; Patrick and Offler, 2001; Milne et al., 2018). Thus, water enters the apoplast of the seed coat (this is not in apoplastic contact with the developing storage tissues) *via* aquaporin water channels located in the plasma membrane of the parenchyma cells and then enters the xylem which exports the water out of the seed (Patrick and Offler, 2001). Various transporters are involved in the transport of assimilates across the plasma membrane of the parenchyma cells of the seed coat into the apoplast that is in contact with the developing storage tissues. Assimilates are then taken up into the storage tissues and various transporters are involved (Milne et al., 2018). Almost nothing is known about the occurrence and localization of these different transporters and channels in the seeds of stone fruits.

The seed coats of both cherry and pea play a role in the interconversion of imported nitrogenous compounds, and one function of these interconversions could be to provide nitrogenous compounds whose subsequent metabolism in the storage tissues produces lower amounts of CO_2_, consumes less O_2_, and does not lead to pH perturbations (Delgado-Alvarado et al., 2007; Walker et al., 2011a). Storage proteins are also accumulated in the seed coat of cherry (Walker et al., 2011a). Enzymes involved in nitrogen metabolism (e.g., PEPCK, PEPC, cytosolic GS, and cytosolic AspAT) are present in cherry seed coat and their abundance g^−1^ FW is highest when both storage proteins are accumulating in the seed coat and a large transfer of nitrogenous compounds to the developing storage tissues is occurring (Walker et al., 2011a). Thus, in the seed coats of stone fruits, it is likely that an extensive metabolism of imported nitrogenous materials occurs.

In the whole seed of various stone fruits, storage proteins are accumulated rapidly during stage II and the beginning of stage III, and a small number of abundant polypeptides account for the bulk of these (Hawker and Buttrose, 1980; Bassi and Ryugo, 1990; Walker et al., 2011a; Famiani et al., 2012). These proteins were accumulated at the same time that proteins were mobilised from the endocarp (Walker et al., 2011a; Famiani et al., 2012). This raises the possibility that proteins stored in the endocarp are translocated to the seed during the hardening of the stone and beginning of stage III, when it accumulates storage proteins (Walker et al., 2011a; Famiani et al., 2012). In black cherry (*Prunus serotina*) seeds, several of these proteins appear to be enzymes (such as amygdalin hydrolase) that have been recruited into the role of seed storage proteins (Zheng and Poulton, 1995). In Japanese apricot, asparagine accounted for about 60–80% of the soluble nitrogenous compounds of the whole seed during stage I, and this percentage decreased to around 35% in ripe fruit. Asparagine (mg g^−1^ FW) content of whole seeds was 6.9 in stage I, 7.5 in stage II, and 1.3 in stage III (ripeness) in 1982 season, and 8.1 in stage I, 4.4 in stage II, and 0.8 in stage III (ripeness) in 1983 season (Kakiuchi et al., 1985). In both the embryo of cherry and whole seed of plum, there is also a large increase in the abundance g^−1^ FW of enzymes involved in nitrogen metabolism (e.g., PEPCK, PEPC, cytosolic GS, and cytosolic AspAT) when storage proteins are being deposited, and this abundance decreases once this accumulation nears completion (Walker et al., 2011a; Famiani et al., 2012). In peach embryos, the bulk of the lipid content is also accumulated during stage III, however, this accumulation continues after that of protein is essentially complete (Bassi and Ryugo, 1990; Walker et al., 2011a; Famiani et al., 2012). Clearly, there must be a massive glycolytic flux from imported sugars to provide pyruvate for this fatty acid synthesis. The seeds of developing stone fruit are white in color, and at least in the case of cherry and plum, do not contain chlorophyll (Walker et al., 2011a). Nevertheless, Rubisco is present and the pattern of changes in its abundance during development mirrors that of enzymes such as GS and PEPC and also that of storage protein accumulation (Walker et al., 2011a; Famiani et al., 2012). Further, it was estimated that the potential amount of activity of Rubisco could be similar to that of some other enzymes involved in storage material deposition (Walker et al., 2011a; Famiani et al., 2012). It has been suggested that Rubisco might function in removing CO_2_ from the tissue and hence reducing its potentially toxic effects (Walker et al., 2011a; Famiani et al., 2012).

In whole seeds of cherry, the contents of malate, glucose, and fructose were similar and around 25 µmol g^−1^ FW when seed FW was rapidly increasing. When seed FW approached its maximum value, the malate content declined to about half this value, whereas, the contents of glucose and fructose increased to 120–160 µmol g^−1^ FW (Walker et al., 2011a). In whole plum seed, each of the content of malate, glucose, and fructose was about 40 µmol g^−1^ FW when seed FW was increasing; and malate content declined when seed FW attained its maximum value (Famiani et al., 2012). In both cherry and plum seeds. sucrose content is much lower than that of glucose and fructose when the seed FW is increasing. These results are consistent with malate, glucose, and fructose contributing to the osmotic potential of the cells in the seeds in order to drive their expansion.

In whole mature seeds of both Japanese plum and sweet cherry the amount of soluble sugars g^−1^ FW is approximately 30% of the amount g^−1^ FW present in the ripe flesh of these fruits (Walker et al., 2011a; Walker et al., 2011b; Famiani et al., 2012). In whole mature cherry seeds, both glucose and fructose are much more abundant than sucrose, whereas, in both mature whole peach and plum seeds sucrose is more abundant than glucose and fructose (Lo Bianco and Rieger, 2002; Walker et al., 2011a; Famiani et al., 2012). In both cherry and Japanese plum seeds, the content of total soluble sugars increases greatly during stage III of development. In cherry seed, sucrose content is low throughout development, whereas in Japanese plum seed, glucose and fructose contents are higher than that of sucrose early in development and sucrose became the most abundant sugar as the seed matures (no data were presented regarding sorbitol contents) (Walker et al., 2011a; Famiani et al., 2012). There are differences in the soluble sugar contents of the embryos of different cultivars of peach. In one cultivar there was a large accumulation of sucrose as the seed matured and its final content was about 12 mg g^−1^ FW, and the contents of sorbitol and hexoses remained low. By contrast, in the embryo of a second cultivar sucrose, glucose and sorbitol were accumulated to final values of 7–15 mg g^−1^ FW (Bassi and Ryugo, 1990).

In whole peach seeds, SuSy activity for stages I, II and III was 1.5, 15.2 and 9.9 μmol g^-1^ FW h^-1^, respectively (Lo Bianco and Rieger, 2002). The values for soluble acid invertase activity at these stages of development were 0.8, 5.2, and 2.7 μmol g^-1^ FW h^-1^, respectively (Lo Bianco and Rieger, 2002). The values for alkaline/neutral invertase activity at these stages of development were 0, 0.8, and 0.5 μmol g^-1^ FW h^-1^, respectively (Lo Bianco and Rieger, 2002). In whole peach seeds at stage III, Morandi et al. (2008) reported an activity of 2 µmol g^−1^ FW h^−1^ for acid invertase, a similar amount of alkaline/neutral invertase activity and an activity of 23-31 µmol g^−1^ FW h^−1^ for SuSY. In the developing seeds and some other storage tissues of some of other plants, it has been suggested that under conditions of low O_2_ status, SuSy is often utilized in catabolizing sucrose in order to provide precursors that are used in the synthesis of storage products (Sturm and Tang, 1999; Morandi et al., 2008; Falchi et al., 2013; Stein and Granot, 2019). In whole peach seeds, the values for NAD-SDH activity at stages I-III were 0.2, 11.2, and 6.5 µmol g^−1^ FW h^−1^, respectively, and the activities of sorbitol oxidase (SOX) at the corresponding stages were 0, 0, and 0.8 µmol g^−1^ FW h^−1^ (Lo Bianco and Rieger, 2002). Very different values were reported in whole peach seeds at stage III by Morandi et al. (2008), where SOX activity was 28–40 µmol g^−1^ FW h^−1^and sorbitol dehydrogenase (SDH) 0.18–0.23 µmol g^−1^ FW h^−1^. Clearly, to understand further the functions of enzymes involved in sugar metabolism in stone fruit seeds, their abundance and locations in the different tissues of the seeds needs to be determined.

## Conclusions and Future Perspectives

Our understanding of the structure, growth and nitrogen and organic acid metabolism of stone fruits is far from rudimentary, and this has allowed an outline of these subjects to be presented in this review. Nevertheless, there are gaps in our detailed understanding of these topics that should be addressed. For example, there is little information available regarding the regulation and localization of enzymes in different tissues and in their different cell types. Further, there is little or no information available regarding certain aspects of the functioning of the vasculature. For instance, how is it possible to supply the different parts of the fruit/seed with a different ratio of sugars to nitrogenous compounds? How are nitrogenous compounds exported from endocarp, and how are these then distributed to other parts of the fruit and seed? Finally, there is the need to further understand the potential process of storage and subsequent release of malate/citrate from the vacuole and its interaction with nitrogen metabolism.

## Author Contributions

All authors contributed to the article and approved the submitted version. However, FF and RW had a major role in the design and writing of the article.

## Conflict of Interest

The authors declare that the research was conducted in the absence of any commercial or financial relationships that could be construed as a potential conflict of interest.
